# Transition: Herausforderungen und Empfehlungen für Jugendliche und junge Erwachsene mit Schizophrenie

**DOI:** 10.1007/s00115-025-01936-1

**Published:** 2026-01-20

**Authors:** Benno Graf Schimmelmann, Alkomiet Hasan

**Affiliations:** 1 Universitätsklinik für Kinder- und Jugendpsychiatrie, Universität Bern, Bern, Schweiz; 2 Klinik für Psychiatrie, Psychotherapie und Psychosomatik der Universität Augsburg, Medizinische Fakultät, Universität Augsburg, BKH Augsburg, Augsburg, Deutschland; 3 Standort München/Augsburg, Deutsches Zentrum für psychische Gesundheit (DZPG), Augsburg, Deutschland; 4 KJP-Hoheluft, Oberstrasse 14B, 20144 Hamburg, Deutschland

**Keywords:** Altersgrenze, Versorgungssystem, Psychose, Früherkennung, Behandlungsübergänge, Age limit, Healthcare system, Psychosis, Early detection, Treatment transitions

## Abstract

**Hintergrund:**

Erste akute Episoden oder schwerwiegende rezidivierende Verläufe einer Schizophrenie treten häufig im Transitionsalter auf – dem Übergang zwischen den kinder- und jugendpsychiatrischen sowie den erwachsenenpsychiatrischen Versorgungssystemen.

**Ziel der Arbeit:**

Erarbeitung von Herausforderungen und Empfehlungen für die Transition von Patientinnen und Patienten mit Schizophrenie.

**Material und Methode:**

Es wurde ein narrativer Review durchgeführt unter Einbezug der aktuellen S3-Leitlinie Schizophrenie der Arbeitsgemeinschaft der Wissenschaftlichen Medizinischen Fachgesellschaften (AWMF).

**Ergebnisse:**

Die leitliniengerechte Früherkennung und Behandlung der Schizophrenie stellt bereits unabhängig von der Transition eine Herausforderung dar. Empfehlungen zur Transition betreffen für Kliniken und Niedergelassene Elemente der Erkrankung selbst, der Versorgungs- und Weiterbildungsstruktur und -kultur sowie der Gestaltung und Finanzierung von Behandlungsübergängen.

**Diskussion:**

Die aktualisierte 2025 publizierte S3-Leitlinie Schizophrenie enthält zahlreiche Empfehlungen für eine koordinierte Behandlung in der Transitionsphase, hält jedoch in großen Teilen an der Altersgrenze von 18 Jahren fest. Für eine optimale Versorgung im Transitionsalter sind spezialisierte Früherkennungs- und Frühinterventionszentren erforderlich. Eine bidirektionale Flexibilisierung der Altersgrenze zwischen den Versorgungssystemen sowie eine überlappende Behandlung in der Transitionsphase erscheinen in bestimmten Fällen sinnvoll.

**Zusatzmaterial online:**

Die Online-Version dieses Beitrags (https://doi.org/10.1007/s00115-025-01936-1) enthält eine tabellarische Übersicht der für den Altersbereich unter 18 Jahren zugelassenen Antipsychotika nach S3-Leitlinie.

## Abstract (en)

**Background:**

First acute episodes or severe recurrent courses of schizophrenia often occur in the age of transition, the transition between the pediatric psychiatric and the adult psychiatric care systems.

**Objective:**

Examination of the challenges and recommendations for the transition of patients with schizophrenia.

**Material and methods:**

A narrative review was carried out taking the current S3 guidelines of the Association of the Scientific Medical Societies in Germany (AWMF) on schizophrenia into consideration.

**Results:**

The guideline-conform early detection and treatment of schizophrenia is already a challenge independent of the transition. Recommendations on the transition period for clinics and private service providers concern elements of the disorder itself, the structure of the healthcare system and specialized training and culture, and the concepts and financial compensation for treatment transitions.

**Conclusion:**

The updated German S3 guidelines on schizophrenia published in 2025 include numerous recommendations for coordinated treatment in the transition phase but in most parts maintain the age cut-off at 18 years. For optimal care in the age for transition, access to specialized early detection and intervention centers is essential. An overlapping treatment during the transition phase and a bidirectional flexibility of age limits within healthcare systems appear to be beneficial in certain cases.

Die Schizophrenien gehören zu den psychischen Erkrankungen, deren erste Episode im Alter der Transition zwischen Kinder- und Jugendpsychiatrie und -psychotherapie (KJPP) und Erwachsenenpsychiatrie und -psychotherapie (EPP) erkannt und behandelt werden müssen. Sowohl das (Früh‑)Erkennen als auch die frühzeitige leitliniengerechte Behandlung stellen hohe Herausforderungen an die beiden Versorgungssysteme und die Transition zwischen diesen Systemen. Entsprechend hat dieser komplexe Behandlungsbedarf der Schizophrenien international zu altersübergreifenden Früherkennungs- und Behandlungskonzepten geführt.

## Infobox 1

Der Fokus dieser Arbeit ist die Schizophrenie. Im Zusammenhang mit der Früherkennung werden Patient:innen mit nichtaffektiven Psychosen betrachtet. Ersterkranktenprojekte als Modell für gelungene Transitionskonzepte wiederum fassen unter Psychosen sowohl affektive als auch nichtaffektive Psychosen zusammen.

## Hintergrund

Die Schizophrenien sind mit einer Lebenszeitprävalenz von knapp 1 % und von 4,0 auf 1000 Einwohnerinnen und Einwohner (Median; [10, 13]) eine häufige psychiatrische Erkrankung. Die Ersterkrankungsrate ist in der 1. und 2. Lebensdekade am höchsten, und ca. 15 % aller Schizophrenien beginnen vor dem 18. Lebensjahr. Komorbiditäten wie Sucht, Aufmerksamkeitsdefizit‑/Hyperaktivitätsstörung (ADHS), Zwangserkrankungen oder Autismusspektrumstörungen sind häufig.

Die Früherkennung und Frühintervention bei psychotischen Störungen (und insbesondere der Schizophrenien) (siehe Infobox 1) zielen zum einen auf das Erkennen eines klinischen Risikosyndroms („clinical high risk for psychosis“, CHR-P) bei hilfesuchenden Jugendlichen und Erwachsenen, das ein höheres Risiko für eine manifeste Psychose anzeigt. Bereits unabhängig vom Psychoserisiko ist das klinische Risikosyndrom mit einer kurz- und langfristigen Beeinträchtigung in Lebensqualität und Funktionsniveau assoziiert und sollte entsprechend gemäß der im Vordergrund liegenden Störungen behandelt werden [2]. Zum anderen zielt die Früherkennung auf die Reduktion der Dauer der unbehandelten Psychose (DUP), also eine frühe leitliniengemäße Behandlung der manifesten Psychose.

Angenommen wird, dass etwa 20 % aller Menschen mit Schizophrenie eine vollständige Wiederherstellung des ursprünglichen Funktionsniveaus und Remission der Symptome erreichen [2]. Neben den das akute Bild dominierenden Positivsymptomen treten Negativsymptome und kognitive Beeinträchtigungen häufig akut und postakut auf und sind prognostisch ungünstig. Entwicklungsaufgaben im Jugend- und jungen Erwachsenenalter (z. B. Schulabschluss, Berufsfindung, Ablösung/Autonomie, Beziehungen, Sexualität) werden durch Symptomatik und Verlauf der Schizophrenie häufig langfristig verzögert oder nicht vollständig bewältigt. Daher ist ein interdisziplinäres und integriertes Behandlungskonzept dringend erforderlich, das Symptomremission und Verbesserungen im Funktionsniveau und Lebensqualität zum Ziel hat. Schon innerhalb der KJPP und EPP stellt diese Aufgabe eine hohe Herausforderung dar. Der Übergang zwischen KJPP und EPP ist dann ohne ein strukturiertes Konzept der Transition (siehe Infobox 2) eine Sollbruchstelle, die zu Therapieabbrüchen, Chronifizierung und Rückfällen führen kann. Dies trifft vor allem auf Fälle mit schweren und therapieresistenten Verläufen zu.

### Infobox 2

Transition ist definiert als Übergang zwischen den Versorgungssystemen Kinder- und Jugendpsychiatrie und -psychotherapie (KJPP) und Erwachsenenpsychiatrie und -psychotherapie (EPP), üblicherweise im Alter zwischen 16 und 25 Jahren.

## Unterschiede zwischen früh beginnender (< 18 Jahre) und im Erwachsenenalter beginnender Schizophrenie

Die Symptomatik von VEOS („very early onset schizophrenia“, < 14 Jahre, ca. 3 % aller Schizophrenien) und EOS („early onset schizophrenia“, 14–18 Jahre, ca. knapp 13 % aller Schizophrenien) und Schizophrenien mit Beginn im Erwachsenenalter (AOS, „adult onset schizophrenie“) ist ähnlich [2, 3]. Prinzipiell weisen EOS und insbesondere VEOS jedoch im Vergleich zu AOS mehr entwicklungsneurologische Auffälligkeiten und ein niedrigeres prämorbides soziales Anpassungsniveau auf sowie mehr Komorbiditäten insgesamt und speziell mehr Autismusspektrumstörungen, mehr kognitive und Negativsymptome und unspezifische emotionale, antisoziale und aggressive Verhaltensweisen bei Erstvorstellung [3]. Die Prognose von VEOS und in geringerem Ausmaß auch EOS ist schlechter als die von AOS [3] Fehldiagnosen bei (V)EOS sind häufig und können zu einem verzögerten Behandlungsbeginn (längere DUP) führen. Aufgrund der Phänomenologie bei (V)EOS mit langer DUP wird die Erkrankung nicht selten mit typischen Krisen in der Adoleszenz verwechselt. Im Kindes- und Jugendalter ist weiterhin nach S3-Leitlinie das Vollbild der Schizophrenie nach ICD-10 (International Statistical Classification of Diseases and Related Health Problems 10) von nicht hinreichend spezifischen Symptomclustern (z. B. formale Denkstörungen, Gedankenlautwerden, Stimmenhören, ausgeprägtes Misstrauen mit sozialem Rückzug, Hypersensibilität, Beeinträchtigungserleben) zu trennen [2]. Im Erwachsenenalter ist insbesondere die Abgrenzung zu substanzbedingten psychotischen Störungen (v. a. Cannabis, Amphetamine) mit Persistenz der Symptomatik nach Konsumstopp herausfordernd.

Die Behandlung von (V)EOS und AOS ist prinzipiell gleich mit einem besonderen Fokus auf die antipsychotische Pharmakotherapie und die kognitive Verhaltenstherapie. Zu beachten sind aber die unterschiedlichen Entwicklungsaufgaben und der besondere Stellenwert der Familie bei (V)EOS sowie die höhere Rate von Nebenwirkungen unter Antipsychotikatherapie (mehr extrapyramidalmotorische Nebenwirkungen, Früh- und Spätdyskinesien, Gewichtszunahme und metabolische Veränderungen sowie Sedation und Prolaktinerhöhungen; [2]). Weiterhin ist der unterschiedliche Zulassungsstatus der Antipsychotika zu würdigen, der nicht kongruent mit positiven Wirksamkeitsnachweisen aus Studien und Metaanalysen im Kindes- und Jugendalter ist. Während im Erwachsenenalter in Deutschland in etwa 30 Antipsychotika zugelassen sind, sind es im Kindes- und Jugendalter definiert als < 18 Jahre 6 Antipsychotika (Tabelle e1 im Onlinezusatzmaterial; [2]).

Positive Wirksamkeitsnachweise liegen vor für Aripiprazol, Haloperidol, Olanzapin, Quetiapin, Paliperidon und Risperidon [12]. Bis 2025 gab es in den Leitlinienempfehlungen zur antipsychotischen Pharmakotherapie bis auf die Beschränkungen aufgrund der Zulassung einen relevanten Unterschied zwischen KJPP und EPP: Clozapin bei Therapieresistenz hatte in der KJPP eine schwache und in der EPP eine starke Empfehlung. 2025 wurde dieser Unterschied zugunsten einer starken Empfehlung auch in der KJPP aufgehoben [2]. Die Empfehlungen für psychotherapeutischen Interventionen sind für (V)EOS und AOS gleich, allerdings fehlen Vergleichsstudien.

### Früherkennung

Bei der Früherkennung psychotischer Störungen sind folgende Unterschiede zu beachten: Zunächst treten bei Kindern häufiger „atypische psychotische Symptome“ auf. Dies sind vor allem Halluzinationen, die mehrere Sinneskanäle betreffen können (szenische Halluzinationen), lebendiger und flüchtiger sind, einen Zusammenhang mit einem Lebensereignis oder einer emotionalen Belastung haben, häufig einen sekundären Krankheitsgewinn mit sich bringen und schneller remittieren [7]. Risikosymptome für eine psychotische Störung umfassen altersübergreifend abgeschwächte psychotische Symptome (APS) und kurze limitierte psychotischen Symptome (BLIPS), die als „ultra-high risk“ (UHR) zusammengefasst werden. In vielen Studien werden zusätzlich die kognitiven Basissymptome (COGDIS) aus dem Basissymptomkonzept verwendet [11]. UHR und COGDIS definieren ein klinisches Risikostadium für die Entwicklung einer psychotischen Störung (CHR-P). Im Kindes- und Jugendalter ist CHR‑P weniger spezifisch für einen Übergang in eine manifeste Psychose und weniger häufig mit Beeinträchtigungen des Funktionsniveaus verbunden [14]. Diese Datenlage führte zu Leitlinienempfehlungen, die zu mehr Vorsicht und Zurückhaltung bei der Kommunikation eines Psychoserisikos und vor allem bei einer Antipsychotikabehandlung von CHR‑P im Transitionsalter aufrufen [2].

## Herausforderungen der Schizophrenie Behandlung in der Transition

In Tab. 1 sind zunächst die Herausforderungen an eine leitliniengerechte Behandlung von Menschen mit einer Schizophrenie insgesamt, also unabhängig von der Transition, dargestellt und im zweiten Teil die für die Transition relevanten Probleme.

**Tab. 1:** Herausforderungen in der Schizophreniebehandlung in der Erwachsenenpsychiatrie und -psychotherapie und der Kinder- und Jugendpsychiatrie und -psychotherapie

Herausforderungen wo?	Herausforderungen welche?
Allgemein	– Lange Wartezeiten oder keine freien Behandlungsplätze in KJPP und EPP im ambulanten Sektor – Später/fehlender Zugang zu nicht-pharmakologischen Maßnahmen (KVT; Familien/Suchttherapie; Supported Employment) – Veränderter Suchtmittelkonsum (v.a. Cannabis und Amphetamin) und höherer Level an Aggressivität gegen sich selbst und andere – Fehlende Angebote für Früherkennung und Frühbehandlung mit Behandlungskontinuität durch Case-Management, Psychose-Knowhow des gesamten Helfersystems, integrierter sozialpsychiatrischer Behandlung, ambulant vor stationär
KJPP	– Relativ seltene Erkrankung in diesem Bereich führt zu vermindertem Wissen – Dadurch Fehldiagnosen, später Einsatz von Antipsychotika und speziell Clozapin – (Angst vor) Antipsychotika Off-Label Use – Fehlende Daten zu Depot-Antipsychotika, Elektro-Krampftherapie
EPP	– Zu geringer und zu wenig budgetierter Einbezug von Familie/Angehöriger – Fehlende Verfügbarkeit von spezialisierten Zentren – Fehlende Verfügbarkeit von EKT-Plätzen – Sorgen vor dem Einsatz von Clozapin
Erkrankungsbedingte Transitionsprobleme	Häufig erste *Akutphase* genau im Transitionsalter – mit geringer/fluktuierender Krankheitseinsicht – mit Verunsicherung Betroffene/Angehörige, Selbstwerte/Selbstwirksamkeit ⭣ Hoher *postakuter* Bedarf an Behandlungskonstanz und Integration von Behandlungselementen – durch Negativ- und kognitive Symptomatik – durch verzögerte/gescheiterte Entwicklungsaufgaben (Autonomie, Beziehung, Schule/Beruf) – durch häufige Komorbiditäten (Sucht, Depression, Soziale Phobie, ADHS, Autismusspektrumstörungen) – durch Nebenwirkungen von Antipsychotika mit schwankender Bereitschaft zur Rezidiv Prophylaxe Bedarf bei *CHR-P* an Behandlungskonstanz und integrierte Behandlung – unterschiedlich, aber bei Menschen mit Psychose-nahem Erleben ähnlich hoch
Transitionsprobleme durch Behandlungssystem	*Klinik*: – Kulturunterschiede (KJPP traditionell ganzheitlicher, mehr Familien-Einbezug; EPP sozialpsychiatrische Tradition) – Fehlende altersübergreifende Früherkennungs- und behandlungsangebote für psychotische Störungen – Fehlende Flexibilisierung des Transitionsalters und fehlende überlappende Behandlungsmöglichkeit – Fehlende gemeinsame Weiterbildung – Fehlende Sturkturierung von Informationsfluss und Abläufen in der Transition *Niedergelassene*: – Flexibilisierung Transitionsalter nur in KJPP vorhanden, nicht in EPP – Fehlende Finanzierung von Transitions-Mehraufwand und überlappender Behandlung – Speziell fehlende Finanzierung sozialpsychiatrischer Leistungen für ältere Adoleszente in EPP – Fehlende Strukturierung von Informationsfluss und Abläufen in der Transition – Fehlende Netzwerke zur integrierten und altersübergreifenden Behandlung von Psychosen und deren Finanzierung

*CHR-P* Clinical High Risk for Psychosis, *EPP* Psychiatrie/Psychotherapie, *KJPP* Kinder und Jugendpsychiatrie/Psychotherapie, *KVT* Kognitive Verhaltenstherapie

## Empfehlungen zur Transition von KJPP zu PP

Eine ausführliche Übersicht zu Empfehlungen zur Transition bei Schizophrenie mit Bezug auf europäische Versorgungssysteme liefert die Arbeit von Arango et al. [1]. Allgemeine Transitionsempfehlungen sind in dieser Ausgabe von *Der Nervenarzt* (siehe Poustka et al.) und bei Fegert et al. [6] und spezifisch für Schizophrenie bei Karow et al. [8] zu finden. Aus diesen Empfehlungen wurden die folgenden Empfehlungen unter Würdigung der neuen S3-Leitlinie Schizophrenie abgeleitet.

### Sofort umsetzbare Empfehlungen

#### Schnittstellenmanagement

Schnittstellen zwischen KJPP und EPP sollten an den Übergängen im stationären und ambulanten Bereich strukturiert werden. Strukturiert werden sollten die benötigten Informationen und der Ablauf des Übergangs, beides schlank und effektiv, um mehr Bürokratie zu vermeiden. Nötig sind Informationen zum Verlauf der Symptomatik inklusive Komorbiditäten, zur pharmakologischen Therapie (u. a. Ansprechen, Dauer bis zur Response/Remission, Nebenwirkungen, Nonresponse/Therapieresistenz), zum aktuellen Helfersystem und zum Funktionsniveau. Arango et al. [1] empfehlen mit Recht, Menschen mit Schizophrenien nach Risiko für Behandlungsabbrüche zu stratifizieren und den Aufwand für den Transitionsprozess entsprechend zu erhöhen. Der Ablauf des Übergangs stellt bereits in vielen Regionen ein großes Problem dar, weil im Niedergelassenenbereich zu wenig Kolleginnen und Kollegen neue betroffene Personen aufnehmen können. Hier empfehlen sich regionale Netzwerke, die für beide Seiten eine Win-win-Situation darstellen können. Die KJPP könnte beispielsweise Kinder betroffener Personen der EPP aufnehmen und im Gegenzug die EPP erwachsen werdende Menschen mit einer Schizophrenie aus der KJPP. Niedergelassene benötigen für die Zusammenarbeit mit Kliniken klare Ansprechpersonen, einfache Aufnahmemusterdokumente und verlässliche und rechtzeitige Übergaben schriftlich und telefonisch (je nach Risikostratifizierung) poststationär. Bezüglich der Off-label-Problematik in der Anwendung von Antipsychotika wird ein gemeinsames Auftreten von Klinik und Niedergelassenen vor den Krankenkassen empfohlen, um den Einsatz des besten Antipsychotikums auch im Kindes- und Jugendalter durchzusetzen.

Die Koordination des Transitionsablaufs in Kliniken ist aufgrund der größeren Nähe zwischen EPP und KJPP einfacher, sollte aber durch Verantwortliche repräsentiert und im Ablauf standardisiert werden. Gerade in der klinikinternen Transition ist die Stratifizierung des zu treibenden Aufwandes für den Übertritt zwischen den Fachgebieten nach Risiko eines Behandlungsabbruchs oder nach Komplexität der Behandlungselemente sinnvoll (z. B. Koordination des weiteren ambulanten Hilfesystems und Evaluation des Bedarfs an psychosozialer Unterstützung (z. B. „supported education oder employment“), Behandlung von Komorbiditäten, vor allem Sucht).

All diese Empfehlungen erscheinen einfach, sind aber erfahrungsgemäß nur selten konsequent umgesetzt. Konkret wird in der Phase der Transition empfohlen, außer in dringenden Fällen (Nonresponse, relevante Komplikationen) keine Medikamentenumstellung vorzunehmen [1].

#### Therapeutische Beziehung

Die rechtzeitige und angemessene Information von Menschen mit einer Schizophrenie, deren Familien und Vertrauenspersonen sowie dem erweiterten Helfersystem zur Transition ist notwendig. Diese Elemente können in die Psychoedukation im Einzel- sowie Gruppensetting mit und ohne die Einbeziehung von Angehörigen und anderen Vertrauenspersonen integriert werden. Wie bei anderen Störungsbildern auch sind Hoffnungen und Befürchtungen angemessen in beiden Versorgungssystemen anzusprechen. Die Vorbehandler sollten prägnante Informationen zu diesen Themen an den Weiterbehandelnden übermitteln, um Enttäuschungen vorzubeugen und damit die Behandlungsabbruchsrate zu senken. Peer Support wäre eine interessante Option, diesen Übergang zu begleiten, aber aktuell stößt die Finanzierung von Erfahrungsexpertinnen und -experten in Deutschland trotz der Möglichkeit nach PPP-RL (Richtlinie zur Personalausstattung in der Psychiatrie und Psychosomatik) aufgrund der Zurückhaltung der Kostenträger an die Grenzen.

#### Weiterbildung

Regional kann erreicht werden, dass für die Transition relevante Themen ausreichend angeboten werden. Damit Weiterzubildende (aller Fachgruppen) der EPP und KJPP lernen, jeweils die Perspektive des anderen Bereichs einzunehmen, lohnen sich *gemeinsame* Weiterbildungsveranstaltungen. Diese sorgen auch für ein persönliches Kennenlernen der an der Transition Beteiligten und verbessert damit die Kommunikation an der Schnittstelle. Es ist zu erwarten, dass gemeinsame Weiterbildungen zu diesem Thema das Bedürfnis nach altersübergreifenden Behandlungskonzepten steigern und damit die Transition für Menschen mit Schizophrenie flexibler und kompetenter machen. Arango et al. betonen zudem den notwendigen Know-how-Austausch zu den in der EPP erst seit ca. einem Jahrzehnt beachteten Entwicklungsstörungen wie ADHS und Autismusspektrumstörungen, die bei Schizophrenie häufig komorbid auftreten [1]. Darüber hinaus könnten Themen wie der Einbezug der Familien, die sozialrechtlichen Grundlagen der Off-label-Verordnung, entwicklungsbiologische Faktoren (kognitive und Methodenkompetenz nach [Muster-]Weiterbildungsordnung [MWBO]), aber auch konkrete Praxisbeispiele wie z. B. Umgang mit herausforderndem Verhalten von Angehörigen, Beantragung von Unterstützungsangeboten (Handlungskompetenz nach MWBO) Inhalte gemeinsamer Weiterbildung sein.

### Empfehlungen, die spezifische Behandlungskonzepte benötigen

#### Früherkennungsambulanzen

Vergleichsweise einfach ist die Implementierung eines altersübergreifenden Früherkennungszentrums. In diesem werden hilfesuchende Personen auf das Vorliegen eines CHR‑P gescreent, gemonitort und bei Bedarf behandelt. Dieses kann ausgeweitet werden auf die Früherkennung anderer altersübergreifender und komplexer Störungen, die eines besonderen Diagnostik-Know-hows bedürfen, wie Persönlichkeitsstörungen, Autismus und ADHS im Jugend- und jungen Erwachsenenalter. Ein großer Vorteil ist die Zunahme der diagnostischen Kompetenz von KJPP und EPP, komplex kann allerdings die Verzahnung mit den übrigen Angeboten der Kliniken sein. Ziel wäre ein spezialisiertes diagnostisches Kompetenzteam, das sein Wissen an die übrigen klinischen Angebote zurückspielt. Die Transition zwischen KJPP und EPP wäre hiermit klinikintern bestmöglich für Menschen mit CHR‑P gewährleistet. Das bestehende Finanzierungsproblem im Niedergelassenenbereich durch das Verbot der gleichzeitigen Abrechnung sozialpsychiatrischer Behandlungen in der KJPP und der ambulanten Abklärung in einem Früherkennungszentrum muss dabei politisch gelöst werden.

#### Frühbehandlungszentren

Die integrierte und altersübergreifende Behandlung ist eine deutlich aufwendigere, aber notwendige Empfehlung [1, 2]. Gemeint sind niedrigschwellige psychosespezifische Behandlungskonzepte mit Case-Management, integrierten Behandlungsmodulen, häufig mit aufsuchenden Angeboten („assertive community treatment“), dem Vorzug ambulanter gegenüber (teil)stationärer Behandlung, mit Einbezug des Helfernetzwerkes/der Familie und Strategien, Recovery – also ein bestmögliches Funktionsniveau und Lebensqualität – zu erreichen. International haben diese Konzepte eine gute Evidenz für eine niedrigere Rate von Behandlungsabbrüchen, eine höhere Zufriedenheit mit der Behandlung, ein höheres Funktionsniveau und eine bessere Lebensqualität. Allerdings beeinflussen sie den langfristigen symptomatischen Erkrankungsverlauf nur bedingt [5], dies vermutlich vor allem dann, wenn diese Behandlungskonzepte zu kurzfristig angelegt sind. Die S3-Leitlinie Schizophrenie hat hierzu die starke Empfehlung 124 in der Version 4.0 in 2025 aktualisiert [2]. Dazu gehören:

- die leitliniengerechte Pharmakotherapie und Psychotherapie,
- Familieninterventionen,
- Maßnahmen zur Integration auf dem ersten Arbeitsmarkt,
- Möglichkeit zu niederschwelligen Behandlungsangeboten oder aufsuchenden Behandlungen und
- eine verstärkte Zusammenarbeit auf hausärztlicher, fachärztlicher und betriebsärztlicher Versorgungsebene.

Ziele sind dabei die Verkürzung der Dauer der unbehandelten Psychose, der frühzeitige Zugang zu einer phasenspezifischen Behandlung und koordinierte auf Ersterkrankung spezialisierte Diagnostik und multiprofessionelle Behandlung.

Eine Transition gelingt in einem integrierten und altersübergreifenden Frühbehandlungszentrum für Psychosen naheliegenderweise deutlich besser [1]. Ausschließlich eine gemeinsam betriebene Adoleszentenstation in Kliniken ist für Menschen mit Schizophrenie ein guter erster Schritt, greift aber angesichts der notwendigen Integration vor allem ambulanter Angebote zu kurz.

### Empfehlungen, die gesundheitspolitische Maßnahmen oder eine spezielle Finanzierung benötigen

Die oben genannten Frühbehandlungskonzepte benötigen vor allem dann eine andere Finanzierung, wenn das Konzept „ambulant vor (teil)stationär“ in der Behandlung umgesetzt werden soll, aber auch für die Finanzierung aller relevanten Behandlungsmodule. Wie in den Studien zum Hamburger ACT(Assertive Community Treatment)-Modell [9] gezeigt, wird nicht unbedingt eine höhere Finanzierung zur Behandlung benötigt, aber es muss eine Flexibilisierung der ambulanten und (teil)stationären Finanzmittel geben mit Umverteilung von (teil)stationärer Vergütung zugunsten von aufsuchender Behandlung, intensiverer und interdisziplinärer ambulanter Behandlung und Case-Management. Bislang müssen ambitionierte Kliniken Modellprojekte mit sehr hohem Aufwand konzipieren und mit (vielen) Krankenkassen verhandeln, obwohl es genug Evidenz für die Notwendigkeit solcher Behandlungskonzepte für bestimmte Patientengruppen gibt. In vielen Regionen erhalten die Kliniken dann negative Bescheide, sodass die aktuellen Bemühungen Einzelfallprojekte bleiben.

Für die gelingende Transition bei Menschen mit einer Schizophrenie sind *überlappende* Behandlungen zwischen KJPP und EPP klar indiziert und sparen durch Verringerung der Behandlungsabbruchrate sehr wahrscheinlich auch Geld. Konsensuspapiere definieren, dass eine überlappende Behandlung in KJPP und EPP für Schizophrenie von bis zu einem Jahr möglich sein muss, stratifiziert nach Abbruchrisiken und Komplexität des Therapiebedarfs [1]. Dies gilt für den ambulanten Klinik- und den Niedergelassenenbereich. Neben der überlappenden Finanzierung ist auch zu diskutieren, ob es in der EPP nicht eine Finanzierung komplexerer sozialpsychiatrischer Therapieangebote für schwere Störungen wie Schizophrenien geben muss, im Niedergelassenenbereich z. B. die Einführung einer Pauschale für Menschen mit schweren psychischen Erkrankungen mit der Möglichkeit, sozialpsychiatrische Mitarbeitende für Case-Management, aufsuchende Angebote oder spezielle Therapien einzustellen. Auch für diese Konzepte lohnt sich eine möglichst einfach zu definierende Stratifizierung nach Diagnose, Schweregrad, Risiko und individuellen Bedarfen der betroffenen Personen, um den knappen Ressourcen des Gesundheitssystems gerecht zu werden. Ansonsten bergen neue Finanzanreize die Gefahr, dass weniger betroffene Personen unnötig aufwendiger behandelt werden, und die komplexen und schwer erkrankten Menschen mit einer Schizophrenie insbesondere in den vulnerablen Phasen der Transition, des Rezidivs und der Therapieresistenz weiterhin auf der Strecke bleiben.

Neben der überlappenden Behandlung in der Transition sollte im Fall der Schizophrenien auch eine Flexibilisierung des Alters der Transition diskutiert werden. Wenn die Transition nicht streng mit 18 Jahren umgesetzt werden muss, wäre eine Anpassung an die individuellen Bedürfnisse der betroffenen Personen besser möglich. Dies macht umso mehr Sinn, als der Entwicklungsstand, aber auch die Erkrankungsschwere von Adoleszenten mit Schizophrenie sehr variiert. Es gibt Fälle mit schwerer therapieresistenter Schizophrenie oder Iron-triangle-Konstellationen (Schizophrenie, Katatonie und Autismus; [15]), die bereits im Alter < 18 Jahren das Setting der EPP benötigen. Gleichzeitig gibt es Menschen mit einer Schizophrenie und Reifungs- sowie Entwicklungsstörungen, die auch im Alter > 18 Jahre von der KJPP profitieren würden. Ein Beispiel für ersteres ist die KJPP-Empfehlung 118 [2] für die Elektrokonvulsionstherapie in der S3-Leitlinie Schizophrenie. Für Niedergelassene aus der KJPP ist es bereits möglich, bis zum 21. Lebensjahr und in begründeten Einzelfällen auch darüber hinaus zu behandeln. Für Niedergelassene der EPP ist ein früherer Einstieg (unter 18 Jahren) allerdings nicht möglich. In Kliniken ist der Zuständigkeitsbereich nach Alter häufig noch strenger geregelt, was vor allem durch die Vorgaben der Kostenträger zu erklären ist, und erzeugt eine „ruppige“ Transitionen für alle Störungsbilder (Verlegung am 18. Geburtstag). Obwohl eine separate Adoleszentenpsychiatrie und -psychotherapie wie in Australien weitere Transitionsprobleme an den jeweiligen Schnittstellen zur KJPP und EPP erzeugt [4], muss anerkannt werden, dass Adoleszenten in unserem Versorgungssystem eine wenig an ihre Bedürfnisse angepasste Behandlung im Spannungsfeld zwischen den Systemen KJPP und EPP vorfinden. Dies ist insbesondere im stationären Kontext in Bereichen ohne spezialisierte Versorgung der Fall.

#### Forschung

Der Bereich Forschung sei unter „gesundheitspolitische“ Empfehlungen dargestellt, damit Evidenz zu politischer Diskussion und schlussendlich zu einer gesundheitspolitisch umgesetzten Verbesserung der Versorgungsstruktur führt. Modellprojekte für eine gelingende Transition (Schnittstellenmanagement) sind weiter sinnvoll und sollten das Ziel haben, die Behandlungsabbruchrate von Menschen mit Schizophrenie zu reduzieren und letztlich den Verlauf zu verbessern. Außerdem sollte die Machbarkeit (kurze und pragmatische Abläufe und Informationsflüsse) in unterversorgten Regionen und angesichts knapper finanzieller und personeller Mittel besondere Berücksichtigung finden.

Zur Evidenz von Früherkennungs- und Behandlungszentren benötigt es keine grundsätzlich neuen Konzepte für Modellprojekte, weil die Datenlage für die Frühdiagnostik und Frühintervention bei Menschen mit einer Schizophrenie international als ausreichend und evident angesehen werden kann [5]. Allerdings sind Versorgungsstudien zur Bedeutung einzelner Behandlungsmodule unter Einbezug digitaler Angebote und unter Berücksichtigung von Kosteneffektivität im deutschen Versorgungssektor dieser Früherkennungs- und Behandlungszentren weiterhin wichtig.

## Fazit für die Praxis

- Die S3-Leitlinie Schizophrenie (2025) formuliert viele Empfehlungen für eine koordinierte Behandlung in den vulnerablen Phasen der Transition, aber hält sich an die Altersgrenze 18 Jahre.
- Sofort umsetzbare Empfehlungen sind gemeinsame Weiterbildungen, die Strukturierung bürokratiearmer Abläufe und Vorgaben für eine angemessene Kommunikation zur Transition mit den Betroffenen.
- Für die optimale Versorgung von Menschen mit einer Schizophrenie im Transitionsalter werden spezialisierte Früherkennungs- und Frühinterventionszentren benötigt.
- Eine bidirektionale Flexibilisierung der Grenze zwischen Kinder- und Jugendpsychiatrie und psychotherapie (KJPP) und Erwachsenenpsychiatrie und -psychotherapie (EPP) kann die Bedürfnisse der Menschen mit einer Schizophrenie in bestimmten Fällen besser adressieren als es bisher der Fall ist.

## Supplementary Information

Tabelle e1: Tabelle der für den Altersbereich unter 18 Jahren zugelassenen Antipsychotika nach S3-Leitlinie [2]. Details sind der genauen Darstellung in der Leitlinie zu entnehmen. FGAs: Antipsychotika der ersten Generation. SGAs: Antipsychotika der zweiten Generation
