# Supplementary material for: Transition: challenges and recommendations for adolescents and young adults with schizophrenia
Source: Nervenarzt. 2026 Jan 20;97(3):223–31. [Article in German] doi: 10.1007/s00115-025-01936-1 (PMC13714110; doi:10.1007/s00115-025-01936-1)
Supplement: Supplementary file 1 [file 115_2025_1936_MOESM1_ESM.pdf]

Tabelle e1: Tabelle der für den Altersbereich unter 18 Jahren zugelassenen Antipsychotika nach S3-Leitlinie [2]. Details sind der genauen Darstellung in der Leitlinie zu entnehmen. FGAs: Antipsychotika der ersten Generation. SGAs: Antipsychotika der zweiten Generation

**FGAs**		**SGAs**	
**Chlorprothixen**[6]	**≥ 3 Jahre**	**Aripiprazol**	**≥ 15 Jahre**
**Haloperidol**	**≥ 13 Jahre**	**Clozapin**	**≥ 16 Jahre**
**Levomepromazin**[5]	**≥ 16 Jahre**	**Paliperidon**	**≥ 15 Jahre**
